# Simulated Epidemics in an Empirical Spatiotemporal Network of 50,185 Sexual Contacts

**DOI:** 10.1371/journal.pcbi.1001109

**Published:** 2011-03-17

**Authors:** Luis E. C. Rocha, Fredrik Liljeros, Petter Holme

**Affiliations:** 1 IceLab, Department of Physics, Umeå University, Umeå, Sweden; 2 Department of Sociology, Stockholm University, Stockholm, Sweden; 3 Department of Energy Science, Sungkyunkwan University, Suwon, Korea; Penn State University, United States of America

## Abstract

Sexual contact patterns, both in their temporal and network structure, can influence the spread of sexually transmitted infections (STI). Most previous literature has focused on effects of network topology; few studies have addressed the role of temporal structure. We simulate disease spread using SI and SIR models on an empirical temporal network of sexual contacts in high-end prostitution. We compare these results with several other approaches, including randomization of the data, classic mean-field approaches, and static network simulations. We observe that epidemic dynamics in this contact structure have well-defined, rather high epidemic thresholds. Temporal effects create a broad distribution of outbreak sizes, even if the per-contact transmission probability is taken to its hypothetical maximum of 100%. In general, we conclude that the temporal correlations of our network accelerate outbreaks, especially in the early phase of the epidemics, while the network topology (apart from the contact-rate distribution) slows them down. We find that the temporal correlations of sexual contacts can significantly change simulated outbreaks in a large empirical sexual network. Thus, temporal structures are needed alongside network topology to fully understand the spread of STIs. On a side note, our simulations further suggest that the specific type of commercial sex we investigate is not a reservoir of major importance for HIV.

## Author Summary

Human sexual contacts form a spatiotemporal network—the underlying structure over which sexually transmitted infections (STI) spread. By understanding the structure of this system we can better understand the dynamics of STIs. So far, there has been much focus on the static network structure of sexual contacts. In this paper, we extend this approach and also address temporal effects in a special type of sexual network—that of Internet-mediated prostitution. We analyze reported sexual contacts, probably the largest record of such, from a Brazilian Internet community where sex buyers rate their encounters with escorts. First, we thoroughly investigated disease spread in this dynamic sexual network. We found that the temporal correlations in this system would accelerate disease spread, especially at shorter time scales, whereas geographical effects would slow down an outbreak. More specifically, we found that this contact structure could sustain more contagious diseases, like human papillomavirus, but not HIV. These results highlight the importance of prostitution in the global dynamics of STIs.

## Introduction

Spatiotemporal heterogeneities in sexual contact patterns are thought to influence the spread of sexually transmitted infections (STIs). Since epidemics can be a society-wide phenomenon, and sexual contact patterns can have structure at all scales, we need population-level sexual network data to understand STI epidemics. Unfortunately, it is hard to collect sexual contact data on that large a scale. Instead, people have focused on small-scale studies using interviews [1]–[3] or contact tracing [4]–[8], or they have studied larger sample sets using random sampling surveys [9]–[13]. Small surveys and contact tracing risk missing large-scale structures [13] and emergent phenomena. Large-scale surveys, on the other hand, have mainly collected the number of partners, but not the connections between them. An alternative way of gather information about sexual contact patterns, which covers a large number of people and explicitly maps their connections, is to use Internet data. In our study, we used a dataset of claimed sexual contacts between Brazilian escorts (high-end prostitutes) and sex buyers [14]. Contact patterns of commercial sex cannot be generalized to a whole population, but they do contain relevant information that can be used to study possible transmission pathways within a social group. Our dataset has information about the time and location of sexual contacts covering six years and 16,748 individuals.

Sexual contact patterns have temporal correlations both at an individual and at a population level [14]. Much like the network structure, temporal structures may influence epidemics in several ways. For example, consider three individuals, A, B, and C, where B and C are in contact first, and later A and B. Considering the temporal order of the contacts, disease cannot spread from A to C via B, but in a standard static network representation this sequence of events is lost, so C appears reachable from A via B [15]–[19]. A conspicuous temporal structure in human behavior that we also observe in our data is bursts of activity during which people are very active for a limited period at a time [20]. Another example of a temporal structure is the long-term behavioral change in which new individuals enter the system and others leave. These temporal effects result in a heterogeneous distribution of inter-event times [14]. To investigate such temporal effects empirically requires time stamps on the contacts. Internet data sets like ours, as opposed to most above-mentioned data, contain just such information.

Extending epidemiological models to include space is a common step towards inclusion of structure beyond the well-mixed assumption [21], [22]. Geography leaves several imprints on the contact structure and thus on disease spread, making the contact network larger than a random graph in terms of graph distances; it also creates the network clusters corresponding to densely populated areas [14]. These effects stress the importance of network-data sets covering a wide geographic area. Our data set, although just a small fraction of the global sexual networks, probably represents a substantial fraction of the Internet-mediated escort business of Brazil [14].

In this paper, we address the question of how the dynamic contact structure in the contact data of Rocha *et al.* [14] affects epidemic spread in general. For most of our study, we look at spread processes confined to our contact data. Because of the lack of similar data on other types of sexual interaction, it is hard to draw conclusions about the role of the escort business on the spread of STIs in society as a whole. Rather, we investigate the contribution of topological, temporal, and geographic structure on transmission pathways within this specific type of commercial sex. We do, as an example of how our study can be applied to more specific cases, make a crude estimate of the role of our commercial-sex network in the spread of HIV in a population-wide context.

## Materials and Methods

### The empirical sexual network

The web community from which our dataset is obtained is a public online forum openly visible online. The full dataset is available as support information (Dataset S1). It is oriented to heterosexual males (sex buyers), who evaluate and comment on their sexual encounters with female prostitutes (sex sellers), both using anonymous aliases. The posts on the forum are organized by the city location of the encounter and by type of prostitution as defined by price level and mode of acquiring customers (for example, escorts, street sex-workers, brothels). We focus on the escorts section, the most expensive form of prostitution [23] of the forum, mostly because it is better organized than the other sections—each escort is discussed in a unique thread. This forum can straightforwardly be represented as a bipartite network—we connect a sex buyer (one type of node) posting in a forum thread to the escort (another type of node) discussed in the thread. An edge in this network represents one sexual encounter between two individuals. The edges are tagged with the dates of the posts, which we take as an estimate of the time of the sexual encounters, even though the sex buyers often post about several encounters at the same session. Consequently, the order of the posts does not have to be exactly the same as the order of the actual encounters. The dataset covers the beginning of the community, spanning the period September 2002 through October 2008. All in all, 50,185 contacts are recorded between 6,642 escorts and 10,106 sex buyers. Even though the network is spread out over twelve Brazilian cities, these contacts make up a network with a largest connected cluster covering more than 97% of the individuals (see Rocha *et al.* [14] for a thorough analysis of this sexual network). To minimize finite-size effects, we discard the initial 1000 days available in the original data set that correspond to a transient period with fewer users and sparse encounters. One thousand days is an adequate choice, since after that period, the average temporal profile is approximately stationary (see Ref. [14] for details). For better statistical significance, we sample several windows of 800 days. For example, one network sample (of the original network) is obtained by taking all nodes and links that occurred in the period between 1000 and 1800 days; another sample is from the period between 1001 and 1801 days, and so on up to the interval 1200 and 2000 days. The average number of vertices of all windows is *N* = 10,526±145, and the number of contacts (links) is *C* = 27,973±3,612, where ± corresponds to the sample standard deviation.

Apart from the anonymous aliases of sellers and buyers and time stamps, posts also include the buyers' grades of the escorts' performance and information about the types of sexual activity performed during an encounter, divided into three categories: oral sex (with or without condom), mouth kissing, and anal sex. All posts, however, are assumed to report vaginal intercourse (random inspection supports this assumption). In our simulations, for the sake of simplicity, unless otherwise stated, we use all available links and disregard the fact that they possess different levels of risk. Most contacts between a seller and buyer happen only once. By inspection, several users report that next time they buy sex, they prefer a different escort, even if the encounter was graded good. We can expect that not all Brazilian escorts and customers of such are present in the data. Furthermore, posting about an encounter is a low-cost action by the sex-buyer that gives him status in the community, which makes it likely that the reports from most users are quite complete. For most of this paper, we ignore this and study disease spread on a network defined by our data set as it is, which limits our conclusions to effects of temporal structure relative to various other scenarios.

### The network models

A common method of studying correlations in empirical contact data is to compare a network with ensembles, where some properties (like the number of nodes and their degrees) are kept constant and the rest is randomized. In the randomized network versions used in this paper, we conserve the bi-partite structure of the heterosexual network and the number of contacts of each individual.

Diverse network structures can affect disease spread [24]–[26]—one example being clustering (a high density of triangles). Our network has a large number of 4-cycles (the shortest cycle in a bipartite graph), and a pronounced community structure, probably a result of the system being geographically embedded [13], This effect can be studied by randomizing the contact pairs in such a way that we choose two links randomly and swap the respective sex-buyers (we call this new null model *random topological*, RT). We do not alter the time stamps of the links; hence, the time order of the escorts' contacts is preserved. To remove temporal correlations, we choose two links randomly and only swap their time stamps such that the new encounter time is unrelated to the original, but the network structure is conserved (this model is named *random dynamic*, RD). Finally, we make a third randomization, where both the temporal and network structures are removed by swapping the time stamps and contact pairs simultaneously (we call this model *random dynamic topological*, RDT).

To put our results in the context of other levels of epidemiological modeling, we also consider two other contact models—a static network approach and the dynamic network model by Volz and Meyers [18]. The static network (SN) approach considers the network of pairs, with at least one contact over a time interval of 800 days, and assumes that contacts can happen with equal probability over all these links. This approach is common to most network epidemiological studies (e.g., refs. [2], [3], [10]). To compensate for the removal of the time stamps, we assume that each link has a certain probability of being active. This probability is derived from our original network and depends on the number of contacts *C* = 27,973 and number of different partners *K* = 21,813 in the window of *T* = 800 days. Thus, the chance of having a contact active is, for our data, *p*_active_ = *C/KT* = 1.603×10^–3^. The idea of Volz and Meyers's model is that vertices change partners with a probability *p*_change_ while keeping the number of partners fixed over time. This model assumes that a vertex is always connected to someone else; however, in our network, in the interval of 800 days, several vertices have only one or few days during which a connection is active. This means that most of the time they are not in a position to catch a disease. To compensate for this effect, and to allow direct comparison to the simulations on the empirical network, we modify the Volz–Meyers (VM) model to capture the brevity of partnerships in the data. In our formulation of the VM network, each vertex has a chance *p_k_* (proportional to the original number of contacts of the vertex) of being active per day. This assures that over the course of 800 days, each vertex has the same number of contacts as in the original empirical network. For each day, we connect pairs of active vertices randomly (if the number is odd, the remaining active vertex is connected the next day that another active vertex is available). Thus, this network has no temporal correlations. We generate VM graphs with 10,526 vertices—the same as the average number of vertices in the sampled windows discussed above. We obtain the degree distribution from these sampling windows as well and use it to calculate *p_k_*.

### Simulation of epidemics

One can model the spread of sexual infection in various ways to capture the various characteristics of pathogens and contact patterns, and also to serve different aims of explanation and prediction. We explore the effects of temporal correlations on different levels of epidemic modeling. The first disease-transmission model we consider is the Susceptible–Infected–Removed (SIR) model. Where all individuals are initially susceptible; upon contact with an infective, a susceptible becomes infective with probability ρ (probabilities are, unless otherwise stated, per-contact probabilities), and after a fixed time δ, a susceptible changes to the removed state. If δ is larger than the vertex lifetime in the network, we get the limit case known as the Susceptible–Infected (SI) model. In a static network of finite size and non-zero transmission rate, all vertices will eventually become infected in the SI model. This is not necessarily the case in a temporal network, which makes the SI model more realistic in temporal, compared to static, contact networks.

To simulate these models in our empirical network, we first map the sampled network onto a time-ordered list. Each entry in the list is one pair of vertices and the time of the contact. Different contacts between the same pair appear as different entries in the list. Then we divide the list into intervals of 800 days each, as mentioned above. The pairs are ordered according to their times of contact. We select the sex-seller of the first contact of an interval as a source of infection and go through the ordered list infecting a susceptible vertex in contact with an infective vertex with probability ρ. The state of the vertex is updated at each new contact. A way of modeling the fact that the network is connected to a background of sexual contacts would be to include multiple sources of infection. To keep the simulations simple, however, we leave this for future studies. Since our temporal information has a resolution of one day, we do not know the order of contacts within a day. To remove this potential bias, we randomize the order of contacts within a single day 100 times. In line with other studies, and to simplify the model, we assume that both infection and removal (after time δ) are immediate, and the transmission probability is constant.

The SI model is adequate for modeling the early phase of an outbreak over shorter time scales than the duration of the disease. SIR, on the other hand, is appropriate for simulating diseases having a well-defined infectious stage followed by immunity. As an example, we will investigate HIV at a more detailed level than simply SI or SIR. Hollingsworth, Anderson, and Fraser [27] devised a model for HIV-1 infection with a susceptible stage followed by four distinct infective stages of different infectivity—one acute infection of high infectivity (over a time-scale of months) followed by a chronic stage (lasting for years), and another high infectivity stage (some weeks) followed by zero infectivity before death. Since our dataset covers only 1000 days, we can omit the last two stages and arrive at a model characterized by an acute stage of transmission probability ρ_1_ lasting for a time *T*_1_, and a chronic stage of transmission probability ρ_2_. We refer to this as SI_1_I_2_ model. Strictly speaking, the transmissibility of HIV-1 also depends on gender and other factors such as type of sex and the fact that the viral load transmitted per-contact can spike during the chronic phase because of comorbidities, among other things. A yet more detailed model could also include an age-stratified population, as young infectives tend to influence an outbreak more. Because they are in the network for longer times, they have higher chance to establish more contacts and contribute to transmit the infection [28].

We follow a similar procedure as above to simulate disease spread in the SN and VM networks. For the initial conditions, however, since the probability of being infected should increase with contact rate in case of the empirical networks, we now select the source of infection randomly (for each realization) and proportionally to the number of contacts of the vertex. This procedure compensates for the fact that in the empirical network, high degree nodes are necessarily selected more than once as a source of infection. This is because, on average, the chance of an individual's being active at a certain moment is proportional to that individual's number of contacts. The state of the vertex is updated after all vertices have been considered. We run the algorithm 30,000 times to obtain averages for these models.

A key quantity is the fraction of infected vertices Ω (*the outbreak size*). If the time evolution is not explicitly stated we refer to Ω at the end of the sampling time window (800 days). We also run simulations 50 times over different initial conditions to calculate the average values.

## Results

### SI model simulation

**Figure 1:**
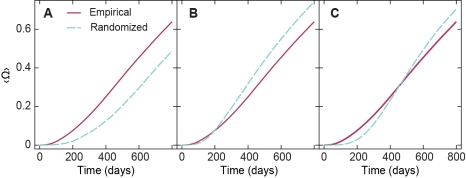
Temporal and topological correlations effect on epidemics. In A–C, we plot the time evolution of the fraction of infected vertices 〈Ω〉. The curves correspond to SI epidemics in the original network (full line) and in its randomized versions: panel A represents swapping time stamps (RD); B shows rewiring of the edges and keeping the sellers' time correlations (RT); and panel C depicts simultaneous randomization of time stamps and edges (RDT).

A straightforward way of investigating the effects of the temporal and topological structure of contact patterns is to remove different types of correlations by randomization (see Section The network models). In Figure 1, we investigate effects of the time ordering of contacts by using the SI model with ρ = 1 and compare the simulated epidemics in the original network with the epidemics in the three different randomized versions of it. In Figure 1A, we see that an infection spreads much more slowly in the RD network model, reaching fewer than 50% of the individuals compared to more than 60% in the original network. Thus, correlations in the order in which the contacts occur speed up disease spread. More concretely, one such tendency is that individuals tend to be intensely active over a period of time followed by idle periods. When the time stamps are randomized (RD model), this tendency disappears such that the presence of individuals in the system is now, on average, longer and the contacts less frequent. The average time, between an individual's first and last active period of, increases from 170.9±0.1 days in the original network to 337.5±0.1 days after randomization. In addition to correlations in the temporal order of contacts, the topology of the sexual network can also influence epidemics [3], [6]–[9], [18]. In Figure 1B, we compare the evolution of epidemics in the empirical network with the RT network model. The evolution of the fraction of infected individuals 〈Ω(*t*)〉 seems to grow slowly, at least during the initial 200 days; afterwards, the topologically randomized network yields more rapid and pervasive outbreaks (Figure 1B). The more rapid initial epidemic spread in the original network results from the high clustering of contacts within cities. Finally, considering both the temporal and topological information randomized (RDT model), the curve (evolution of the epidemics, Figure 1C) is in between those of Figure 1A and Figure 1B. The fraction of infected vertices increases slowly during the initial 300 days, but not more slowly than in the RD scenario in Figure 1A. Later it increases more rapidly and by the end of the sampling period reaches about 70% of the individuals (a little less than in the RT scenario in Figure 1B, but still, larger than in the original network).

**Figure 2:**
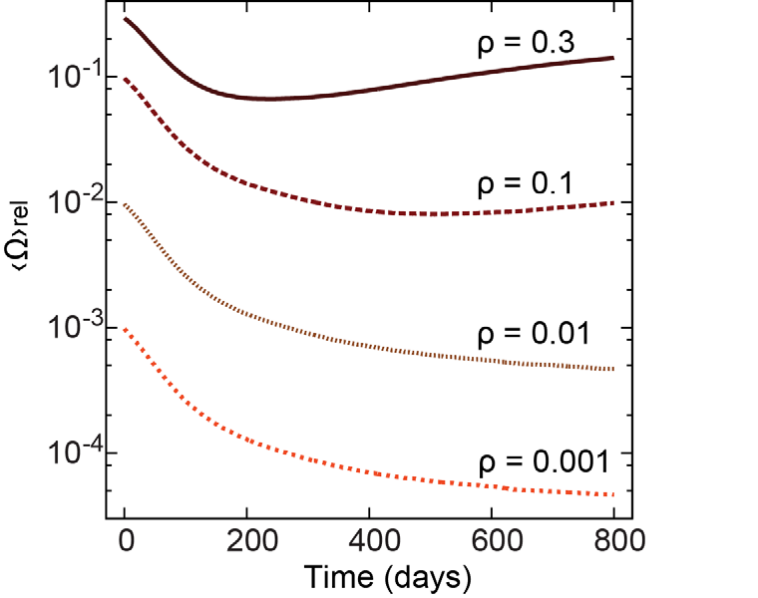
Evolution of the infection for low transmission probabilities in the SI model. The panel shows the evolution of 〈Ω〉_rel_, the number of infected vertices for lower transmission probabilities (0.001≤ρ≤0.3) relative to the number of infected vertices when we use the maximum transmission probability (ρ = 1). The ordinate is in log-scale.

The limit of high transmission probability ρ = 1 does not reflect actual STI contagion; more realistic values lie in the range 0.001≤ρ≤0.3 [27]–[28]. In Figure 2, we present 〈Ω〉rel = 〈Ω_ρ_〉/〈Ω_ρ = 1_〉, the average number of infected vertices (for probabilities ρ) relative to the number of infected vertices when the maximum transmission probability is used (ρ = 1). The relative number of infected vertices decreases within the initial 100 days and afterwards reaches a minimum for higher transmission probabilities while continuing to decrease slowly for lower rates. The minimum, which corresponds to the time lag of secondary infections, is more pronounced for lower ρ-values. The fact that the curves are fairly constant for times longer than 200 days, that is, that they converge to limiting values, is an indication that our results for the ρ = 1 case hold for other transmission probabilities as well, that is, the time-ordering effects are stronger than the fluctuations from the stochasticity of the contagion process. For lower ρ-values, the curves decrease monotonically, which indicates the existence of an epidemic threshold somewhere between, ρ = 0.01 and ρ = 0.001, which we investigate more cautiously below.

**Figure 3:**
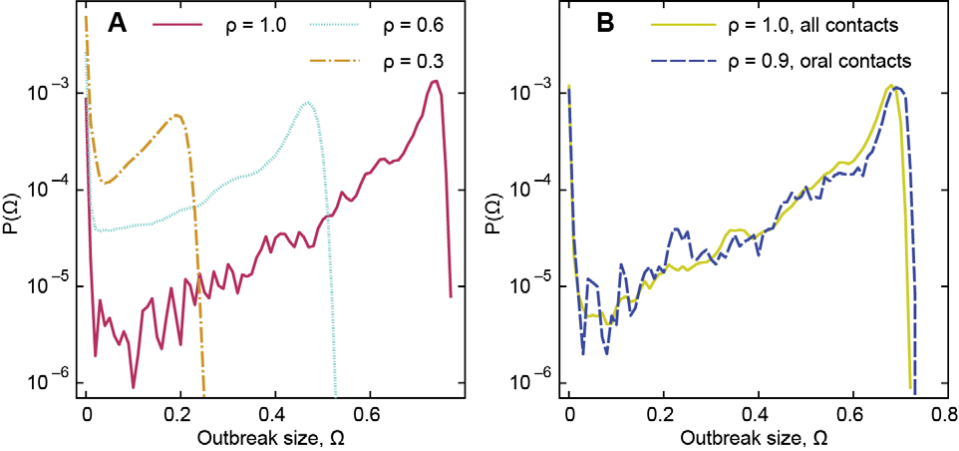
Comparison of the outbreak size distributions for different contagion pathways. In panel A, we plot the probability distribution *P*(Ω) of the outbreak sizes Ω for different transmission probabilities, in the SI model, for the original network, and in panel B for the network considering only encounters with oral sex without condom, and mouth kissing (ρ = 1 and SI model).

We note that there is a large diversity of outbreaks even for ρ = 1. In Figure 3, we measure the probability distribution *P*(Ω) of outbreak sizes Ω. This, we hypothesize, is a general phenomenon—temporal constraints increase the diversity of outbreaks because they restrict the possible infection paths in the network. There is, however, a local maximum where, for ρ = 1, a fraction of about 0.75 of the vertices gets infected, setting a characteristic outbreak size. This local maximum depends on the transmission probability and decreases for lower values. Another observation is that the outbreak-size distribution becomes less heterogeneous for lower ρ-values. The peak on the very left of the graph indicates that the disease is likely to die out. Note that the network also contains some isolated connected components that, once infected, do not spread the infection to the giant component.

To illustrate the effect of different sexual activities, we show the outbreak size distribution for the original network considering only the encounters that involve oral sex without condom, and mouth kissing (Figure 3B). This specific network has roughly the same outbreak-size distribution (similar shape and scale) as the original network, despite being about half as dense.

**Figure 4:**
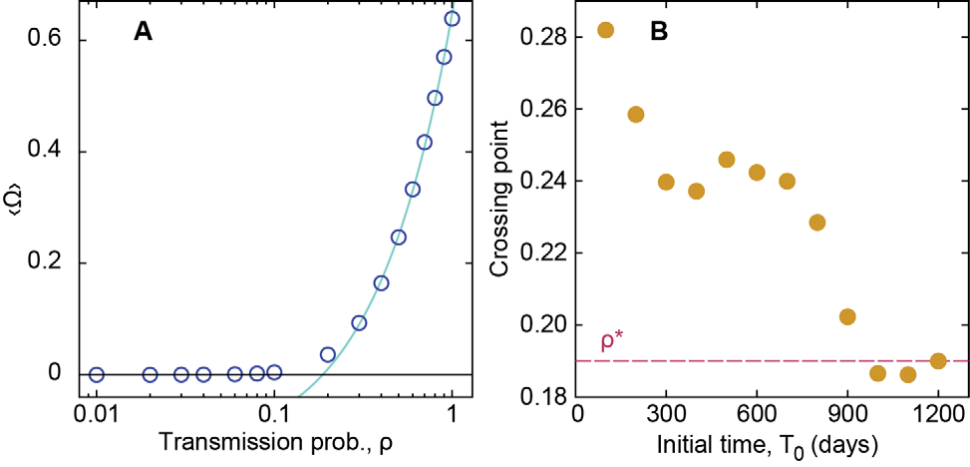
Epidemic threshold for SI model. Panel A displays the average outbreak size 〈Ω〉 as a function of the transmission probability. The line is a linear trend least square fitted to the data in the interval 0.3≤ρ≤1. The abscissa is in log-scale. Panel B shows the threshold ρ-value (estimated by the crossing of the linear fitting and the zero-size outbreak line) as a function of the beginning of the sampling window.

**Figure 5:**
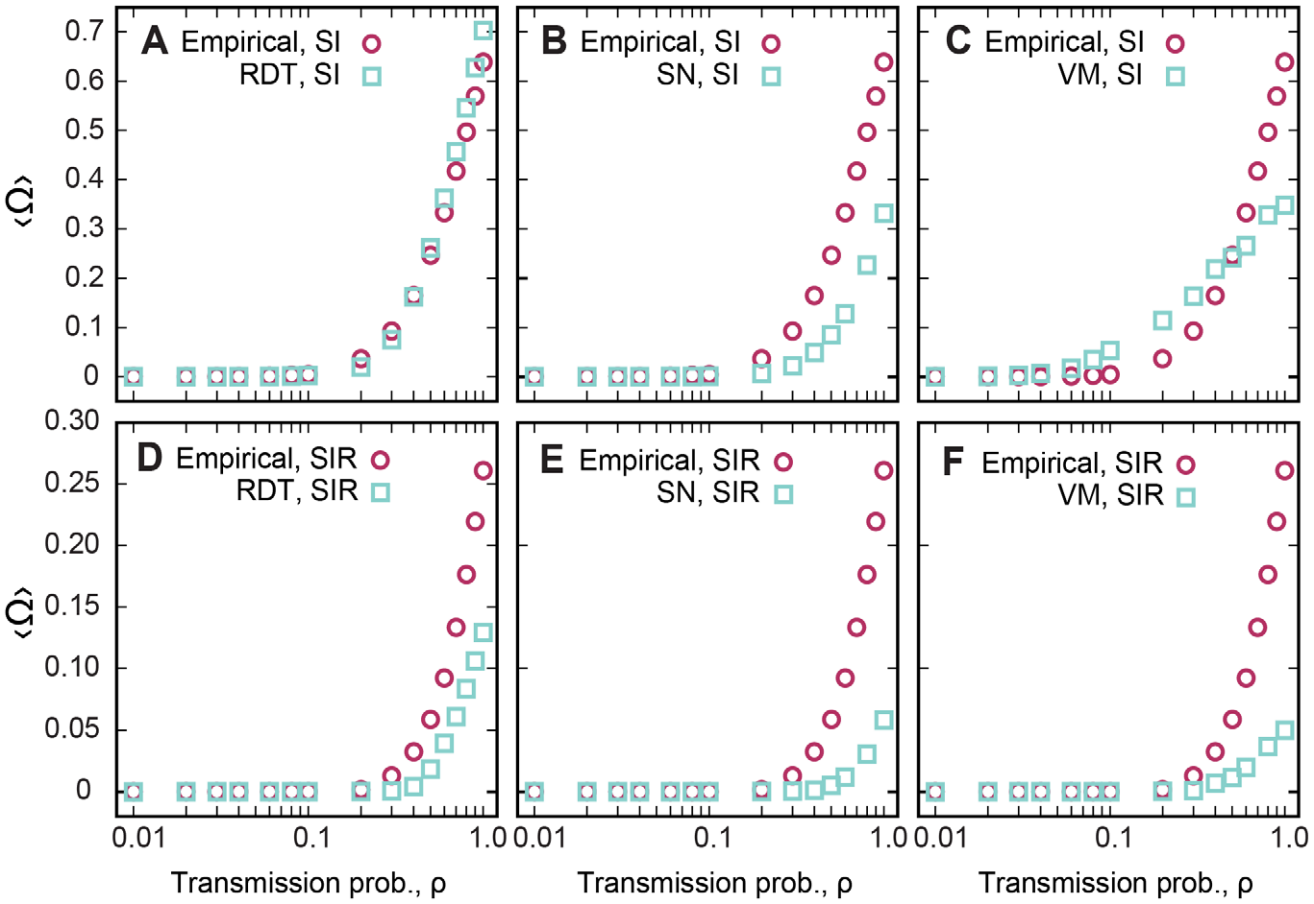
Transmission probability and outbreak size for SI and SIR models. Outbreak size versus transmission probabilities for (A–C) SI and (D–F) SIR epidemic models. Each panel shows the results for the empirical and for a random network. The abscissa is in log-scale.

Returning to our original network, we investigate the effect of varying ρ, and see that the average outbreak size 〈Ω〉 is an approximately linear function of transmission probability (see Figure 4A). From the figure, it is evident that the epidemic outbreak is practically absent for transmission probabilities lower than about 0.19—a *de facto* threshold effect. Looking in more detail, one can see that this threshold effect is due to the fact that the mean value of large outbreaks vanishes and that the number of small outbreaks increases as ρ→0 (cf. Figure 3). To investigate whether this threshold value ρ* is an artifact of the finite-size sampling, we use different sampling windows from the complete dataset and see whether the threshold values converge to a common value for different starting points in the dataset. The threshold values should converge to a limit value as the network structure trends toward a steady state. Each window represents the same duration of time (800 days), but simply starts at a different time (*T*_0_) in the original data set. We take the crossing point between the fit of the fraction of infected vertices as a function of ρ to a line and the line of zero secondary infections as an estimate of the threshold value. We see in Figure 4B the apparent convergence of the threshold estimates to values at about ρ* = 0.19±0.01 for increasing *T*_0_, which is our estimated threshold value for this contact pattern. This threshold seems slightly smaller for the RDT, but significantly smaller for the SN and VM models (Figure 5A–C).

### SIR simulation results

**Figure 6:**
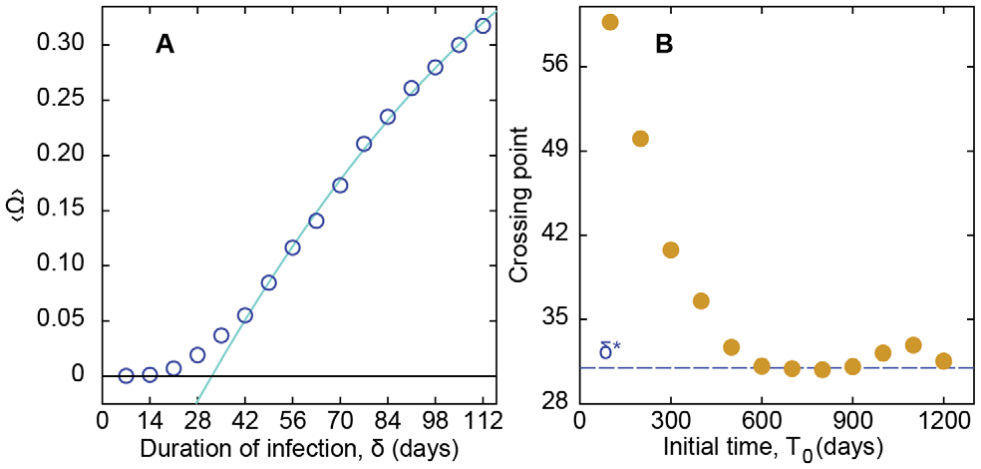
Epidemic threshold for SIR model for ρ = 1. Panel A shows the average outbreak size 〈Ω〉 as a function of the duration of the infective stage *δ*. The line is a second-order polynomial fit to the data in the interval 42 days ≤*δ*≤112 days. Panel B shows the time of such crossing point as a function of the beginning of the sampling window, *T*_0_.

We plot the average outbreak size 〈Ω〉 as a function of the duration of the infective stage δ in Figure 6A. Here, we assume the maximum transmission probability ρ = 1. We proceed to identify estimated threshold values by performing fits of second-order polynomials to the fraction of infected individuals and identify the crossing point with the zero secondary infection line. Performing a similar analysis as for the SI model's transmission probability threshold, but now for the duration of the infective state, we find that the δ-threshold converges to δ* = 31±1 days.

Now, fixing the infective stage to δ = 91 days, which is roughly 3 months and well above our estimated threshold of δ*, we perform SIR simulations for different transmission probabilities and compare the outbreak sizes by using the original network, the randomized version (RDT), a static (SN), and a dynamic network (VM) (Figure 5D–F). For all cases, the thresholds are above ρ* = 0.2, and the final outbreak size is always larger for the empirical network, suggesting that the temporal correlations, the essential difference between the raw empirical contact patterns, and the models accelerate transmission.

### SI_1_I_2_ simulation results

**Figure 7:**
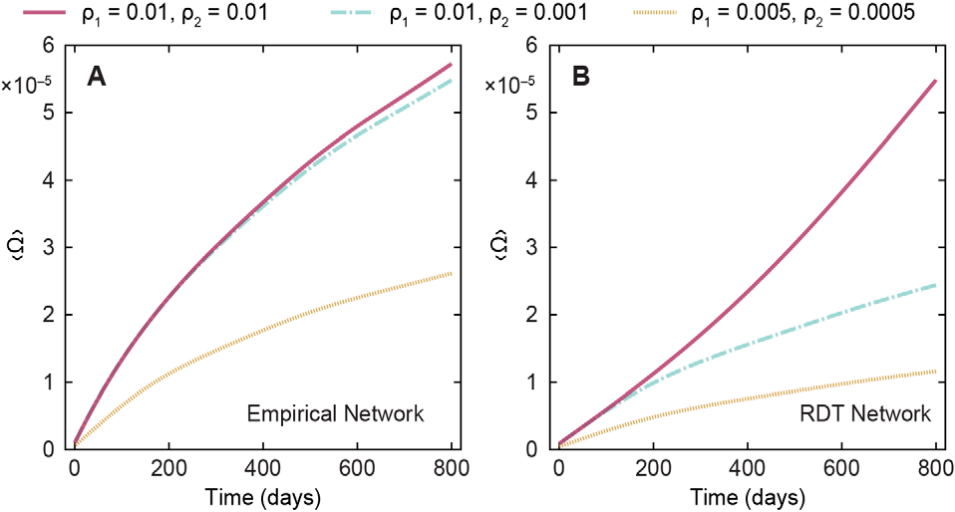
Evolution of the outbreak size for SI_1_I_2_ model. The increase in the number of infected vertices, Ω, using the simulated SI**_1_**I**_2_** model in panel A original network and panel B in a randomized version (RDT network).

Now we turn to the results of the SI_1_I_2_ simulation of HIV spread. We fix the acute infective period at *T*_1_ = 91 days and study some different combinations of estimated transmission probabilities available in the literature for different societies by using lower (ρ_1_ = 0.005 and ρ_2_ = 0.0005) and higher (ρ_1_ = 0.01 and ρ_2_ = 0.001) bounds [27], [29]. In Figure 4A, we see that the threshold transmission probability of the SI model is higher than all these values, so we already know that the SI_1_I_2_ model on the actual data is below the epidemic threshold. In Figure 7, we plot the average time evolution of the outbreak size for both our empirical temporal network (Figure 7A) and the RDT contact model (Figure 7B). The average outbreak sizes are, as expected, very low (a fraction of about 10^–5^ of the population) for both these contact patterns. For the RDT model and ρ_1_ = ρ_2_ = 0.01, the system is just above the epidemic threshold, as can be seen by its convex curve in Figure 7B. A conspicuous temporal feature is that, for the empirical network, the effect of a larger transmission probability of the chronic infection is very small—the ρ_1_ = ρ_2_ = 0.01 and ρ_1_ = 0.01, ρ_2_ = 0.001 curves are almost congruent. For the RDT contact structure, the more homogeneous temporal pattern allows the chronic infection to play a greater role, so these two curves diverge after about 200 days, which is about the average interval between two consecutive contacts.

## Discussion

We simulate the spread of infection in what is probably the largest network of self-reported sexual contacts yet recorded. Our data come from a web community of sex buyers who discuss their encounters with escorts. Although the network is spread out over twelve cities, it is to a large extent connected so that a disease could spread from most parts of the system to most other parts. As with any result based on a subset of a network, we should be cautious about extrapolating our results to the entire society, especially since it is hard to compensate for missing links with the information we have. The escorts in our dataset make up about one percent of all Brazilian sex sellers (of a total of about one million [30]). On the other hand, the escorts are a small fraction of all sex-sellers, and we can tell by the way the average degree (number of partners) converges that the sampling time is longer than an escort's typical duration in the business [14]. Another complication when it comes to generalizing the results of the paper to a society as a whole is that our sexual network is not an isolated system. It is possible that the infection leaves the community and eventually returns through other individuals. In that case, our model would underestimate the impact of the network in the outbreak. Furthermore, commercial sex is not necessarily driven by the same mechanisms as regular sexual interaction. So, since our data is not comprehensive enough to infer the impact of prostitution on disease spread, we focus on how temporal correlations in the empirical data affect results from random and well-mixed models. From studying the SI model with a 100% transmission probability in our sexual network, we conclude that temporal correlations speed up the epidemics, especially in the early phase of superlinear growth. This effect has important implications both for disease modeling, implying that temporal correlations in contact patterns should not be underestimated, and for intervention methods (like targeted vaccination), where temporal structures could potentially be used to detect important individuals. Furthermore, the temporal effects seem to cause well-defined and relatively high epidemic thresholds, unlike studies of model networks with power-law degree distributions [10] where outbreaks can occur of any non-zero transmission probability. For purposes of comparison, in a finite-sized scale-free network and Susceptible-Infected-Susceptible epidemics with recovery μ = 1, ρ_critical_ = 〈*k*〉/〈*k* ^2^〉 (where *k* is the number of different partners of an individual [31]), gives ρ_critical_∼0.043, using our network. The network structure (apart from the contact-rate distribution), on the other hand, slows down outbreaks. Our network has a high density of short cycles and community structure, reflecting the fact that most sex buyers buy sex in one region, presumably their hometown. Both factors, many short clusters and distinct communities are known to slow down diffusion in networks [24]–[26].

Most of our analysis is at a general STI level and indicates that our network is not dense enough to support STI outbreaks for chronic diseases with transmission probabilities lower than ρ = 0.19. The fact that endemic diseases with arguably lower transmission probabilities exist points to the importance of the background sexual contacts. Because of the incompleteness of our data, this does not completely exclude the possibility of escort prostitution as a reservoir of STIs, but it points to a more complex picture. In the support information (Text S1), in a crude assessment of our dataset contribution to the general STI spread, we suggest that it would only affect the degree-correction of *R*_0_ of STIs by a few percent. We also exemplify how temporal structures can affect the spread of a specific pathogen, HIV-1, by simulations of a refined compartmental model. The simulation results indicate that our empirical network alone cannot sustain an outbreak of HIV-1. In general agreement with empirical research [32], [33], our results suggest that pathways (like unsafe man-to-man sex, or intravenous drug use) other than commercial sex are needed to explain the endemic state of HIV epidemics in Brazil [34]. The other studies are, however, from countries other than Brazil; however, they are inconclusive if not controversial [35], [36].

We believe that the study of temporal aspects of contact patterns is, in general, a promising direction for the future. We intend to investigate how far our conclusions can be generalized to other types of cultures, other forms of commercial sex, and hopefully to non-commercial sexual contact patterns.

## Supporting Information

Dataset S1 We provide the full dataset in .csv format containing the sexual network used in this paper. Specific information about the format of the data is inside the file.
(1.20 MB CSV)

Text S1 Augmenting well-mixed models. We make a short analysis of the contribution of the studied commercial sexual network to epidemics if this network is embedded in a larger network of sexual contacts.
(0.20 MB PDF)
