# Supplementary material for: Simulated Epidemics in an Empirical Spatiotemporal Network of 50,185 Sexual Contacts
Source: PLoS Comput Biol. 2011 Mar 17;7(3):e1001109. doi: 10.1371/journal.pcbi.1001109 (PMC3060161; doi:10.1371/journal.pcbi.1001109)
Supplement: Text S1 — Augmenting well-mixed models. We make a short analysis of the contribution of the studied commercial sexual network to epidemics if this network is embedded in a larger network of sexual contacts. (0.20 MB PDF) [file pcbi.1001109.s002.pdf]

Support Information 2 for the paper:

Simulated epidemics in an empirical spatiotemporal network of 50,185 sexual contacts

Luis E. C. Rocha[1], Fredrik Liljeros[2] and Petter Holme[1,3]

[1]IceLab, Department of Physics, Umeå University, 90187 Umeå, Sweden

[2]Department of Sociology, Stockholm University, 10691 Stockholm, Sweden

[3]Department of Energy Science, Sungkyunkwan University, Suwon 440-746, Korea

1. Augmenting well-mixed models

As discussed in section 2.1 of the main text, our sexual network is not isolated, so to estimate the effects of escort business on the disease spreading in society-wide scale one both needs to estimate how large is the fraction of this type of commercial sexual contacts we actually have recorded, and be able to reproduce the sexual contact dynamics in society. This is of course hard to do accurately. Regarding the missing contacts, we will proceed without them and thus obtain a lower bound of the contribution. To model the sexual contacts in the rest of society we assume a well-mixed scenario where everyone have an equal probability of be in contact with everyone else. We compare the change in the basic reproduction number $R_0 = \rho / \mu$ (where ρ is the transmission rate and μ is the recovery rate)—the number of secondary infections in a completely susceptible population—if our recorded contacts are added to the underlying well-mixed contact pattern. In well-mixed models with identical individuals, $R_0 = 1$ marks the epidemic threshold. So if $R_0 > 1$ there is a chance that an epidemic will break out. In order for this threshold criterion to hold for populations with a distribution of contact rates, one can calculate an effective R_0 by using the multiplicative factor Λ

$$R_0 = \Lambda\frac{\rho}{\mu} = \left(\frac{\sigma^2}{c^2} - 1\right)\frac{\rho}{\mu} \tag{S1}$$

where σ^2 is the variance of the contact rate and c is the average contact rate [1]. This R_0 corrects for the diversity of contact rates, but not for all the temporal heterogeneities. We take an estimate of this factor from Liljeros *et al.* [2] where σ = 1.26±0.04 year^{-1} and c = 1.22±0.07 year^{-1} give Λ_{without} = 0.066±0.001 as the correction factor for the population without the contacts from Internet prostitution. These numbers come from a random sample of Swedes aged 18 to 74 years, and are not expected to be the same for Brazil, in fact, according to Ref. [3], c_{woman} = 1.47 and c_{man} = 2.96 (which should be taken with a grain of salt since it would imply that over one third of the sexual contacts are between two males), for this data we have no information about σ^2. Now we use the same values from our data (covering a fraction of about ν = 0.07% of the inhabitants in this age cohort in the sampled twelve Brazilian cities) and obtain σ_+ = 16.3 year^{-1} and c_+ = 5.0±0.1 year^{-1}, which give Λ_{with} = 0.169±0.001 for the correction factor of the entire Brazilian population including the contacts of our data set. (In this calculation we assume the two types of contacts to be uncorrelated so that the variances are additive.) To investigate the difference Δ between Λ for the cases with and without the claimed contacts,

$$\Delta = \Lambda_{with} - \Lambda_{without} = \frac{\sigma^2 + \nu\sigma_+{}^2}{(c + \nu c_+)^2} - \frac{\sigma^2}{c^2} \tag{S2}$$

we plot Δ as a function of c and σ in Figure S1. The sexual contacts in our data would, in this sketchy calculation, contribute to about 0.12 of change in Λ and thus be rather insignificant. If the type of commercial sex we study was properly accounted for in the estimates of σ and c, then one can rather measure the impact of our data by subtracting it from the background activity. This gives roughly the same result but in the other direction—Λ decreases by a factor 0.11.

REFERENCES

1. Anderson RM, May RM (1991) Infectious diseases of humans: Dynamics and control, Oxford University Press, Oxford.

2. Liljeros F, Edling CR, Amaral LAN, Stanley HE, Åberg Y (2001) The web of human sexual contacts. Nature 411: 907-908.

3. Abdo CHN (2004) Descobrimento sexual do Brasil. Summus Editorial, São Paulo.

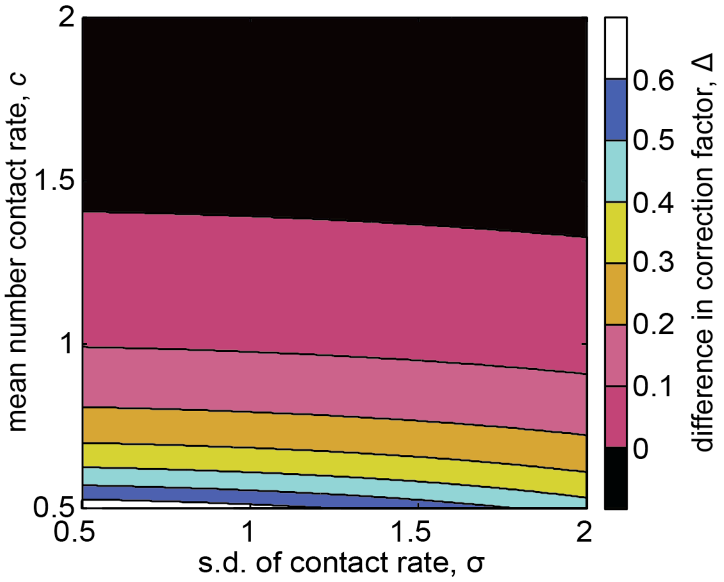

Figure S1. Correction factor for R_0 when adding our contact structure to a sample of the society-wide contact patterns. The difference Δ in the correction factor for R_0 with and without our empirical network as a function of the average contact rate c and the standard deviation of the contact rate σ of the whole population.
